# Development and Characterization of a Polycaprolactone/Graphene Oxide Scaffold for Meniscus Cartilage Regeneration Using 3D Bioprinting

**DOI:** 10.3390/pharmaceutics17030346

**Published:** 2025-03-07

**Authors:** Melike Nur Özder, Aslihan Yelkenci, Mine Kucak, Aylin Altinbay, Cem Bülent Ustündag, Fatih Ciftci

**Affiliations:** 1 Department of Bioengineering, Faculty of Chemical and Metallurgical Engineering, Yıldız Technical University, Istanbul 34210, Turkey; mnozder@gmail.com (M.N.Ö.); cbustun@yildiz.edu.tr (C.B.U.); 2 Department of Pediatric Dentistry, Faculty of Dentistry, University of Health Sciences, Istanbul 34668, Turkey; aslihanzihni@gmail.com; 3 Department of Molecular Biology and Genetics, Yildiz Technical University, Istanbul 34210, Turkey; minekucak@gmail.com; 4 Department of Metallurgical and Material Engineering, Faculty of Chemical and Metallurgical Engineering, Yildiz Technical University, Istanbul 34210, Turkey; aylin.altinbay@yildiz.edu.tr; 5 Health Biotechnology Joint Research and Application Center of Excellence, Istanbul 34210, Turkey; 6 Department of Biomedical Engineering, Fatih Sultan Mehmet Vakıf University, Istanbul 34015, Turkey; 7 Department of Technology Transfer Office, Fatih Sultan Mehmet Vakıf University, Istanbul 34015, Turkey

**Keywords:** 3D bioprinting, graphene oxide, meniscus scaffolds, cartilage, PCL

## Abstract

**Highlights:**

- GO increased the storage modulus of scaffolds from 36.1 Pa to 97.1 Pa.
- Yield shear stress enhanced from 97.2 Pa to 507.1 Pa with GO.
- Optimal mechanical properties achieved with 1% GO: modulus 614 MPa and strength 46.3 MPa.
- GO incorporation increased the melting temperature to 60.78 °C and glass transition to 31.14 °C.
- Roughened scaffold surface improved cell adhesion and cellular distribution confirmed by DAPI staining.
- Antibacterial zones increased against *E. coli* (26.21 mm) and *S. aureus* (15.38 mm).
- Rheological results showed shear-thinning viscosity improvement up to 89.3 Pa·s with GO.
- Elongation at break improved to 10.4% with 5% GO addition.
- GO scaffolds enhanced cell viability to over 100% at 1:8 concentration.
- PCL/GO scaffolds successfully mimicked native meniscus properties with biofunctional and mechanical advantages.

**Abstract:**

**Background/Objectives:** Meniscus injuries represent a critical challenge in orthopedic medicine due to the limited self-healing capacity of the tissue. This study presents the development and characterization of polycaprolactone/graphene oxide (PCL/GO) scaffolds fabricated using 3D bioprinting technology for meniscus cartilage regeneration. **Methods:** GO was incorporated at varying concentrations (1%, 3%, 5% *w*/*w*) to enhance the bioactivity, mechanical, thermal, and rheological properties of PCL scaffolds. **Results:** Rheological analyses revealed that GO significantly improved the storage modulus (G’) from 36.1 Pa to 97.1 Pa and the yield shear stress from 97.2 Pa to 507.1 Pa, demonstrating enhanced elasticity and flow resistance. Mechanical testing showed that scaffolds with 1% GO achieved an optimal balance, with an elastic modulus of 614 MPa and ultimate tensile strength of 46.3 MPa, closely mimicking the native meniscus’s mechanical behavior. FTIR analysis confirmed the successful integration of GO into the PCL matrix without disrupting its chemical integrity, while DSC analysis indicated improved thermal stability, with increases in melting temperatures. SEM analysis demonstrated a roughened surface morphology conducive to cellular adhesion and proliferation. Fluorescence microscopy using DAPI staining revealed enhanced cell attachment and regular nuclear distribution on PCL/GO scaffolds, particularly at lower GO concentrations. Antibacterial assays exhibited larger inhibition zones against *E. coli* and *S. aureus*, while cytotoxicity tests confirmed the biocompatibility of the PCL/GO scaffolds with fibroblast cells. **Conclusions:** This study highlights the potential of PCL/GO 3D-printed scaffolds as biofunctional platforms for meniscus tissue engineering, combining favorable mechanical, rheological, biological, and antibacterial properties.

## 1. Introduction

Meniscus injuries, commonly arising from trauma or degenerative conditions, present a significant challenge in orthopedic medicine due to the tissue’s limited healing capacity [1,2]. The meniscus plays a crucial role in joint stability, load distribution, and shock absorption in the knee, and damage to this tissue often leads to progressive joint deterioration and osteoarthritis. Traditional treatments, such as partial meniscectomy or meniscal repair, offer limited long-term success, especially in patients with extensive damage [3]. Consequently, the field of regenerative medicine has explored novel approaches to restore meniscal function, with tissue engineering emerging as a promising solution [4,5,6,7].

3D bioprinting has become a transformative tool in tissue engineering due to its precision in constructing complex structures tailored to individual anatomical requirements [8,9,10]. By precisely depositing biomaterials and cells layer by layer, 3D bioprinting enables the creation of tissue scaffolds that mimic the natural architecture of the meniscus [11,12,13,14]. This approach not only supports cell growth and differentiation but also allows for customization of mechanical properties to match those of native tissue. Recent studies have focused on the use of various biomaterials to create such scaffolds, with a particular interest in polymers and nanomaterials that offer structural integrity, biocompatibility, and functionality [11,12,15,16].

Polycaprolactone (PCL), a widely used biopolymer, is notable for its biodegradability and mechanical strength, making it suitable for fabricating scaffolds that require long-term stability [14]. However, PCL alone may not provide the necessary bioactivity for effective meniscal tissue regeneration [17,18]. To address this limitation, graphene oxide (GO) has been introduced as an additive to enhance cell attachment, proliferation, and differentiation [19]. GO’s unique properties, including its large surface area, high mechanical strength, and ease of functionalization, make it an ideal candidate for composite scaffold materials in meniscal applications. GO’s conductive nature also supports the transmission of bioelectrical signals, which can further promote cellular activities beneficial for tissue regeneration [20,21,22,23].

In this study, we propose a novel composite scaffold combining GO with PCL, specifically engineered for meniscal regeneration via 3D bioprinting. This scaffold is designed to not only mimic the mechanical properties of the native meniscus but also foster a conducive environment for cellular activity and tissue growth. The introduction of GO into the PCL matrix aims to create a biofunctional scaffold that addresses the limitations of current meniscal repair materials, potentially advancing the treatment options available for meniscal injuries. This study investigates the synthesis, characterization, and bioactivity of the PCL/GO scaffold, aiming to provide insights into its potential application in meniscal tissue engineering.

## 2. Materials and Methods

### 2.1. Preparation of GO and PCL/GO

In this study, GO was synthesized by Hummers’ method [24]. The chemicals used for synthesis such as Graphite flake (mesh size 300), Sulfuric acid (H_2_SO_4_), potassium permanganate (KMnO_4_, 99.9%), phosphoric acid (H_3_PO_4_), and hydrogen peroxide (H_2_O_2_ 30%) were purchased from Merck and Sigma Aldrich (Darmstadt, Germany). In a typical synthesis method, 360 mL concentrated H_2_SO_4_, and 40 mL concentrated H_2_PO_4_ were added to the 1 mL beaker with a large stir bar. The beaker was stirred in an oil bath at 40–45 °C at 200 RPM. Graphite (3 g) was gradually added to the solution. Then, 18 g KMnO_4_ was slowly added, and a deep green color was observed. The mixture was stirred for 16 h at between 40 and 45 °C. Then, the mixture and 400 g of ice were mixed in a 2 L beaker. To the mixture, 3 mL of 30 wt% H_2_O_2_ was added, which changed the color of the mix to yellow. The mixture was poured into the 50 mL centrifuge tubes. Then, the mix in the centrifuge tubes is centrifuged at 3000 RPM for 45 min, and the liquid in the tubes. After the first centrifuge, the mixture was sequentially washed once with distilled water and three times with concentrated HCl (10 wt%) at 3000 RPM for 45 min. Finally, they were washed thrice with ethanol at 3000 RPM for 15 min [25].

To prepare the Poly(ε-caprolactone) PCL solutions, a solvent mixture consisting of acetic acid and formic acid in a 9:1 volume ratio was utilized. The solvents used were 100% anhydrous acetic acid (ISOLAB, Eschau, Germany) and 98–100% formic acid (EMSURE ACS, Reag., Darmstadt, Germany), ensuring high purity. The solvent mixture was prepared by mixing 9 mL of acetic acid with 1 mL of formic acid at room temperature under ambient conditions. PCL with an average molecular weight of 80,000 g/mol (Sigma-Aldrich) was added to the prepared solvent mixture in varying mass concentrations of 5%, 10%, and 20% (*w*/*v*). The PCL-solvent mixtures were allowed to stir at room temperature for 24 h to ensure complete dissolution of the polymer. This approach ensured the homogeneity of the resulting solutions used in subsequent analyses.

GO was added to the PCL solutions at 1%, 3%, and 5% (*w*/*w*), relative to the PCL content (Table 1). The GO was gradually introduced into the PCL solutions under continuous stirring to ensure uniform dispersion. The mixtures were stirred for an additional 6 h at room temperature, followed by ultrasonication for 1 h to prevent agglomeration of the GO particles and achieve a homogeneous polymer matrix. The resulting PCL/GO solutions were stored under ambient conditions and used for subsequent analyses or fabrication processes.

### 2.2. Fabrication of 3D Bioprinted Scaffolds Using PCL/GO Solutions

The 3D printing process was carried out to produce four different scaffold compositions at 50%, 60%, 70%, and 80% infill rates. The 3D-printed scaffolds were fabricated by using a modified GBA 3D printer (Biomaterials and Nanomaterials Laboratory (BİORGİNE Labs), Department of Biomedical Engineering, Fatih Sultan Mehmet Vakıf University, İstanbul, Turkey), Department of Biomedical Engineering, Fatih Sultan Mehmet Vakıf University), which utilized a fused deposition modeling (FDM) system. It included computer-aided design (CAD) technology using a heatable build plate. A digital syringe pump was connected to a 3D printer to control the flow rate of solutions feeding into a syringe with a 0.5 mm nozzle diameter. We fabricated 3D-printed scaffolds using conditions in build plate temperature of 38 °C, a flow rate of 0.2 mL/h, and 0.03 mm distance between the needle and platform.

## 3. Physical Properties and Characterizations

### 3.1. Rheological Characterization

All rheology was performed on an Anton Parr MCR 302 (Graz, Austria) rheometer equipped with a parallel plate configuration (50 mm or 8 mm diameter) at 37 °C, keeping the normal force constant at 0 N. During the optimization, in a typical rheological test for gelation kinetics (i.e., evolution of the storage modulus (G′) and loss modulus (G) as a function of time; time sweep), hydrogels were spread on the bottom plate at 37 °C. The top plate was immediately lowered to a plate separation of 0.5 mm, and the measurement was started. A frequency of 5 Hz and a strain of 1% were applied to minimize interference with the gelation process and to keep the measurement within the linear viscoelastic region. The normal force was also kept constant at 0 N. The gel point was determined by the crossover between G‘ and G′.

### 3.2. Mechanical Properties

A tissue analyzer (Stable Micro Systems, Godalming, UK) was used to evaluate the mechanical properties, including tensile strength and elongation at break, of the designed 3D-printed materials. Composite materials were prepared as cylindrical films with a diameter of 6 mm and a height of 100 mm for compressive tests and as films measuring 5 cm in length, 3 cm in width, and 3 mm in thickness for tensile tests. The composite materials were tested at a 10 mm/min speed up to a maximum strain of 80%. Each sample was measured in quadruplicate. The compressive modulus and Young’s modulus of the bioactive composite materials were calculated using the slope of the initial linear section of the strain-stress curves corresponding to 0–25% strain. To assess the flexibility and recoverability of the composite materials, cyclic compressive tests were performed for four consecutive compressive cycles up to 70% strain, following the same rate. Each sample was tested in four repetitions [26].

### 3.3. Uniaxial Tensile Tests

All uniaxial tensile tests were conducted using an M100-1CT Testometric (Guangdong, China) machine equipped with a 1 kN load cell. Specimens (n = 8–10) were tested either immediately after preparation (i.e., following overnight gelation) or after immersion in culture medium for 1, 7, 14, and 21 days. A preload force of 0.1 N was applied, and each test was performed at a compression rate of 5 mm/min. Each sample was subjected to 98% tension to determine the final compressive stress and strain. For cyclic compression tests, each sample was subjected to 30% tension at a rate of 5 mm/min for both loading and unloading phases. Data were analyzed using Wintest version 4.55.0 analysis software.

### 3.4. Morphological Properties

The morphological properties of the samples were analyzed using a scanning electron microscope (Hitachi SU3500 T2, Graz, Austria). The SEM was operated in secondary electron mode at 5 kV, and further image analysis was conducted at a voltage of 10 kV. We ensured conductivity was applied to all samples, as polymers inherently lack this property.

### 3.5. FTIR Analysis

FTIR spectroscopy was conducted to analyze the functional groups present in the produced composite materials. The characterization studies were performed using a Bruker Alpha FTIR (Karlsruhe, Germany) device. The spectral range from 4000 to 400 cm^−1^ was utilized to identify and analyze the functional groups within the samples.

### 3.6. DSC Analysis

Differential scanning calorimetric (DSC) analysis was performed with a Diamond DSC (Shelton, CT, USA). The sample (~5 mg) was placed in an aluminum container and sealed and heated above the melting temperature (Tm) (60 °C) of PCL at 10 °C min^−1^.

### 3.7. In Vitro Swelling and Degradation Test

The swelling and degradation behaviors of 3D composite scaffolds were investigated for PCL, PCL/GO (1%), PCL/GO (3%), and PCL/GO (5%) scaffolds with 3 mg samples. Swelling behavior was investigated at 0, 1, 2, 4, 8, 12, 16, 20, 24, 48, 72, and 96 min and calculated according to Equation (1). The degradation behavior was investigated for 5, 10, 15, 30, 45, and 60 min and calculated and plotted according to Equation (1).

(1) $$\mathrm{Swelling}\;\mathrm{rate}\;(\mathrm{SR})=\frac{W_{w}-W_{d}}{W_{d}}\times 100$$

The values of wet and dry weight were indicated by *W_w_* and *W_d_*, respectively.

(2) $$\mathrm{Degradation}\;\mathrm{rate}\;(\mathrm{DR})=\frac{W_{w}-W_{d}}{W_{d}}\times 100$$

For the swelling ability test of 3D tissue scaffolds, the scaffold samples are first weighed in the dry state, and the dry weight (*W_d_*) is obtained. They are then immersed in phosphate buffer saline (PBS) at a constant temperature of 37 °C for a fixed time interval. Afterwards, excess water is removed with tissue paper, and the wet weight (*W_w_*) is evaluated. The mass swelling ratio (SR) is calculated using Equation (1). For the frostbite test of 3D tissue scaffolds, the scaffold samples are first weighed dry, and the dry weight (*W_d_*) is obtained. They are then immersed in PBS at a constant temperature of 37 °C for a fixed time interval, and after drying at room temperature for 24 h, the wet weight (*W_w_*) is weighed. The mass degradation rate (DR) is calculated using Equation (2).

### 3.8. Antibacterial Analysis

The pathogens used were obtained from Fırat University, Faculty of Veterinary Medicine (Elazığ, Turkey). Bacterial studies were carried out at the BİORGİNE Laboratory, Department of Biomedical Engineering, Fatih Sutan Mehmet Vakıf University. The pathogens used for antibacterial assays consisted of Gram-negative bacteria *Escherichia coli* (ATCC 25922, Manassas, VA, USA) and Gram-positive bacteria *Staphylococcus aureus* (ATCC 25923, Manassas, VA, USA). A disk diffusion test was performed to determine the antibacterial activity of the 3D meniscus scaffolds. *E. coli* and *S. aureus* suspensions were collected from 18 h nutrient broth cultures, adjusted to 0.5 McFarland standard turbidity (1.5 × 10^8^ CFU/mL) and diluted to the desired bacterial density (1:10). Mueller–Hinton agar plates were inoculated with 0.1 mL of bacterial suspension (1.5 × 10^6^ CFU/mL). The 3D meniscus scaffolds were sliced into x (3), y (2), z (1) mm thickness and placed in bacteria-coated Petri dishes after UV sterilization for 2 h. The plates were incubated at 37 °C for 24 h, and the zones of inhibition around the disks were measured with a digital micrometer. Four separate groups were prepared to select the optimum 3D meniscus scaffolds, and the tests were performed three times.

### 3.9. Cytotoxicity

In vitro cell viability was assessed using the indirect MTT test. L929 mouse fibroblast cell line was used for the biological characterization of the 3D meniscus scaffolds. Before the experiment, the 3D meniscus scaffolds were cut into pieces of 1 cm × 1 cm, UV-C sterilized for 20 min on both sides, and incubated in sterile tubes with the complete medium (DMEM-low glucose, FBS (10%, *v*/*v*), and penicillin-streptomycin (1%, *v*/*v*)) at 37 °C for 24 h. L929 cells were seeded in a 96-well plate at a density of 1 × 10^4^ cells/mL per well and incubated at 37 °C with 5% CO_2_ for 24 h. Sterile scaffolds were incubated in the medium for 24 h, and scaffold extracts were obtained. After 24 h of incubation, L929 cells were treated with different concentrations of scaffold extracts at the ratios of (1:1), (1:2), (1:4), and (1:8). Different concentrations of scaffold extracts were prepared by diluting with the complete medium. After treatment, 10 µL of an MTT (Gold Biotechnology^®^, St Louis, MO, USA) solution was added to each well, followed by incubation for three hours. The wells were then aspirated, and 100 µL of DMSO was added to each to dissolve formazan crystals, followed by incubation for 30 min at room temperature. Absorbance was measured at 570 nm using a microplate reader (Biotek, Dallas, TX, USA). Optical density was measured with a multilayer reader at 450 nm, and cell viability was calculated using the following Equation (3):

(3) $$\mathrm{Viability}\;(\%)=(\frac{Optical\;density\;(OD)\;of\;treated\;cells}{OD\;of\;control\;cells})\times 100$$

L929 cells were treated with scaffold extracts at specified concentrations for 24 h and then washed with PBS. A DAPI staining solution was added and incubated in the dark for 5 min. After removing the dye solution, the cells were washed 2–3 times with PBS and observed under a fluorescence microscope.

### 3.10. Cell Adhesion

This study investigated the adhesion and growth of cells on membranes. A mouse fibroblast cell line L929 was grown in a DMEM medium supplemented with 10% FBS, 1% L-glutamine, 1% penicillin, and streptomycin in a humidified incubator with 5% CO_2_ and 100% relative humidity at 37 °C. The 3D scaffold samples were cut into 1 cm × 1 cm pieces. After UV-C sterilization, they were placed in a 24-well medium, and L929 cells were seeded at a concentration of 5 × 10^4^ in 1 mL of the medium per sample. The scaffold samples were incubated at 37 °C for 24 h and 48 h to allow cells to adhere to the scaffold samples. After 48 h of incubation, the growth medium was discarded, and the cells were fixed with 2.5% glutaraldehyde. The fixed cells were washed with PBS and then dehydrated with ethanol (30%, 50%, 70%, 90%, and 100%). Visualization of cell adhesion in the samples was performed by scanning electron microscopy [27].

### 3.11. Statistical Analysis

All the statistical analyses of the data were performed through ANOVA using the GraphPad Prism version 8 software (GraphPad Software Inc., San Diego, CA, USA). The values were given as means ± standard deviation (SD), and the statistical differences were analyzed by *t*-tests. In all cases, *p* < 0.05 and *p* < 0.0001 were considered statistically significant.

## 4. Results and Discussion

### 4.1. Rheological Analysis

The rheological parameters of the prepared hydrogels such as storage modulus (G′), loss modulus (G″), Shear-Thinning Viscosity, or tan δ (damping factor) are shown in Table 2.

While the G′ value of the PCL hydrogel was 36.1 Pa, this value increased significantly as the GO content increased. In the PCL/GO hydrogel containing 5% GO, the G′ value reached about 97.1 Pa. This indicated that GO greatly strengthened the elastic structure of the material [28,29]. On the other hand, the value of G″ increased similarly. It increased from 8.7 Pa for PCL to 34.4 Pa for 5% GO. This increase revealed that GO improved the viscoelastic properties and increased the shear resistance. The viscosity increased significantly with the increase in GO concentration [30,31,32]. The shear-thinning viscosity of PCL was measured as 19.1 Pa.s, and the shear-thinning viscosity of 5% GO was measured as 89.3 Pa.s. This indicates that GO increases the resistance to flow by integrating into the matrix. The shear rate of the PCL hydrogel was 8.7 s^−1^, while it was measured as 49.8 s^−1^ in 5% GO. Yield shear stress of the PCL hydrogel was 97.2 Pa, while it was observed as 507.1 Pa in 5% GO. This proved that GO makes the structure more durable.

### 4.2. Mechanical Analysis

**Figure 1:**
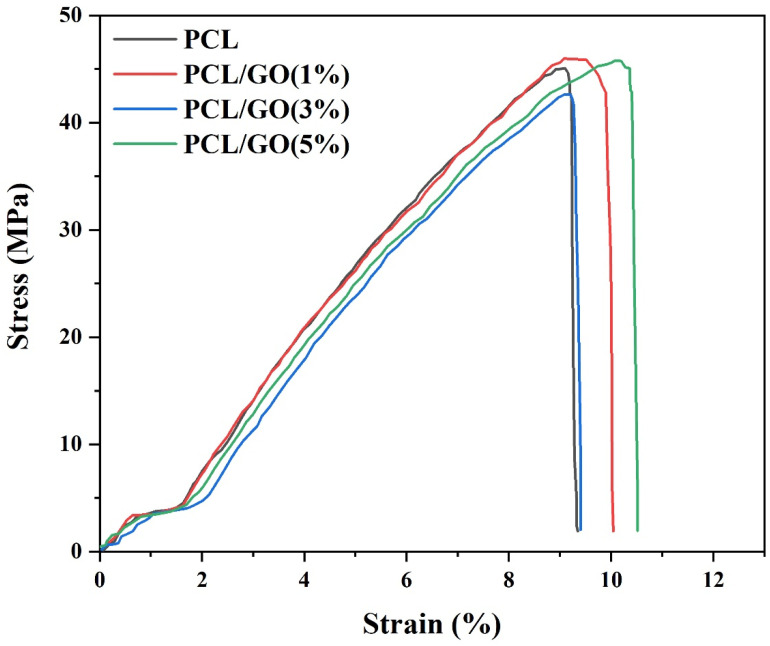
Stress–strain curves of 3D-printed PCL and PCL/GO meniscus scaffolds with varying GO concentrations (1%, 3%, and 5%).

In the mechanical test results for 3D meniscus scaffolds, as the PCL/GO ratio increases (1%, 3%, and 5%), a decrease in the elastic modulus value was observed as 614.1, 592.1, and 588 MPa, respectively (Table 3). The GO additive reduces the elastic modulus, making the material slightly more flexible. The high surface area and flexibility of GO may have slightly reduced the material’s stiffness. However, this may be advantageous for flexible and load-bearing structures such as the meniscus. The PCL/GO (1%) 3D meniscus scaffold showed a slightly higher ultimate stress of 46.3 MPa than pure PCL. However, for PCL/GO (3%), this value was observed as 43 MPa. For PCL/GO (5%), it increased again and reached 46 MPa. It was observed that GO increased the strength at low ratios (1%) but lost its optimal effect at 3% and stabilized again at 5% (Figure 1). When GO was added, the elongation at break increased to 9.2% (PCL), 9.9% (GO 1%), 9.3% (GO 3%), and 10.4% (GO 5%), respectively, compared to pure PCL.

The 5% GO additive reached the highest elongation capacity of the material. The increase in the elongation at the break of GO at the 5% level showed that the material became more elastic and its deformation capacity increased. Regarding mechanical results, PCL/GO (1%) offers a balanced profile regarding the elastic modulus, strength, and elongation. This ratio may be ideal for applications where mechanical properties are critical, such as the meniscus. The curves demonstrate the influence of the GO content on the elastic modulus, ultimate stress, and strain at the break of the scaffolds, highlighting the mechanical performance suitable for meniscus tissue engineering applications.

In similar studies, 3D artificial meniscus-mimicking PCL-agarose (Ag)-gelatin methacrylate (GelMA) hydrogels were designed. Compressive moduli of PCL, PCL-Ag, and PCL-GelMA were 11.5 ± 1.1, 8.4 ± 2.2, and 10.0 ± 1.9 MPa; tensile moduli of the constructs were 30.8 ± 6.6, 27.4 ± 3.8, and 28.1 ± 6.9 MPa. It was observed that the addition of Ag and GELMA hydrogels to the PCL hydrogel and its 3D design caused a decrease in its mechanical properties [33]. In similar studies, 3D scaffolds were designed from PCL-biomimetic coating of chitosan/ECM hydrogel for use in meniscal injuries. The compressive modulus of 3D PCL scaffolds was measured as 5.65 MPa [14].

### 4.3. SEM Analysis

**Figure 2:**
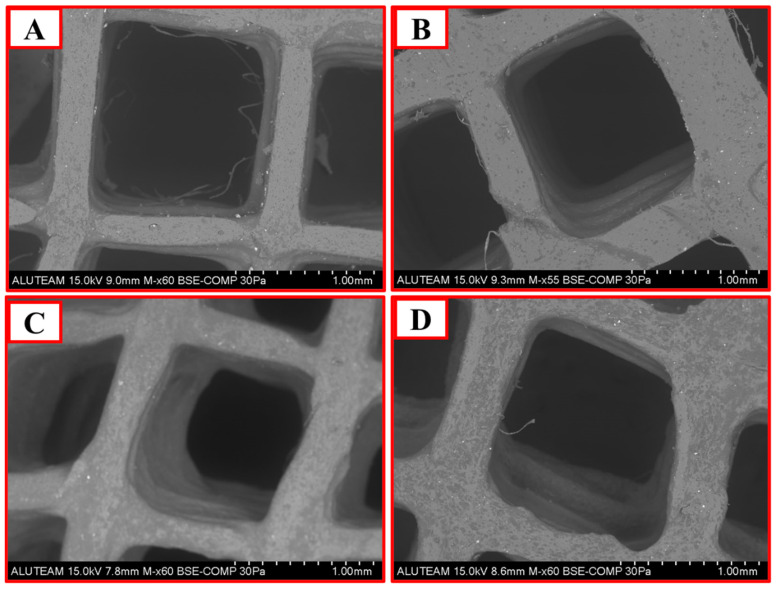
SEM images illustrate the surface morphology and structure of (**A**) PCL, (**B**) PCL/GO (1%), (**C**) PCL/GO (3%), and (**D**) PCL/GO (5%) 3D meniscus scaffolds.

According to morphological analyses of the PCL scaffold, the pores (pore sizes ~1 mm) were observed as a regular and homogeneous structure (Figure 2A). The uniform surface morphology reflects the high workability and controllable properties of pure PCL. In the PCL/GO (1%) scaffold, slight irregularity was observed on the surface with GO doping (Figure 2B). This may improve biocompatibility and cell adhesion. The pore structure (pore size ~2 mm) is regular, but a more pronounced heterogeneity is observed in the walls. This change in the surface may slightly increase mechanical strength while creating a more favorable environment for biological integration. The PCL/GO (3%) scaffold showed more pronounced microstructural irregularities on the surface with an increasing GO content (Figure 2C). The connections between the pores appear sharper and clearer, which may provide favorable effects on mechanical load bearing. The pore sizes remained constant at about 1 mm, but the shape of the pores gained a sharper and more distinct geometry. This can improve the structure stability and the favorable environment for the arrangement of cells. The 5% GO doping created significant irregularity on the surface (Figure 2D). This may favorably affect the attachment and proliferation of cells to the scaffold. However, excessive surface modifications may cause weaknesses in mechanical strength. Pore sizes increased to around 2 mm, and irregularities in the pore walls increased. The density of micro- and nanoscale structures on the surface may give positive results regarding biological performance. As the GO ratio increases in 3D meniscus scaffold design, a noticeable change in surface morphology occurs. This benefits cell attachment, proliferation, and biological integration [34].

### 4.4. DSC Analysis

**Figure 3:**
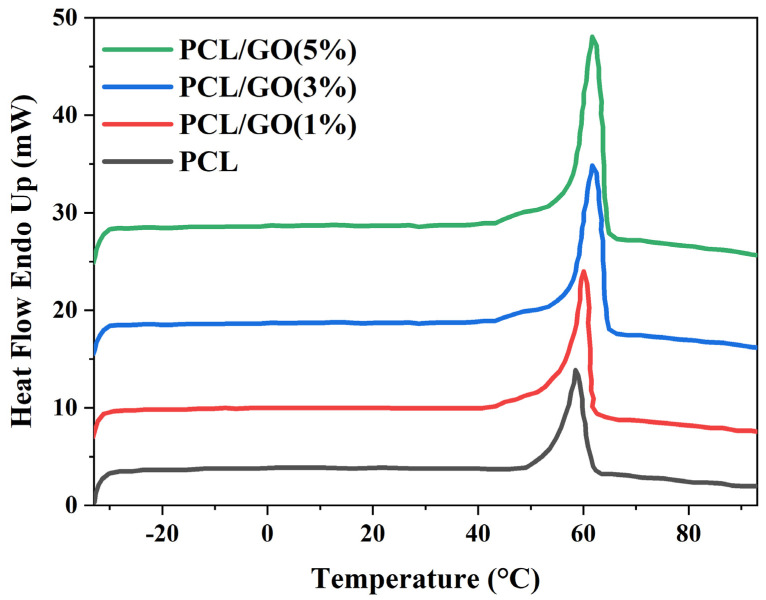
Differential scanning calorimetry curves of 3D-printed PCL and PCL/GO meniscus scaffolds with varying GO concentrations (1%, 3%, and 5%).

The peak temperatures of PCL, PCL/GO (1%), PCL/GO (3%), and PCL/GO (5%) 3D scaffolds were 58.46, 60.03, 61.67, and 61.67 °C, respectively (Figure 3). This small increase in peak temperature may indicate that the incorporation of GO into the PCL matrix slightly improves the thermal stability. The presence of graphene oxide showed that the melting peak was slightly enlarged and higher. This suggests that GO may influence the crystallization behavior of the polymer and possibly increase the order of the crystalline structure. It can be observed from the DSC curves that GO changes the melting temperature and thermal behavior of the PCL matrix. The addition of GO generally improves the thermal stability [35,36].

### 4.5. FTIR Analysis

**Figure 4:**
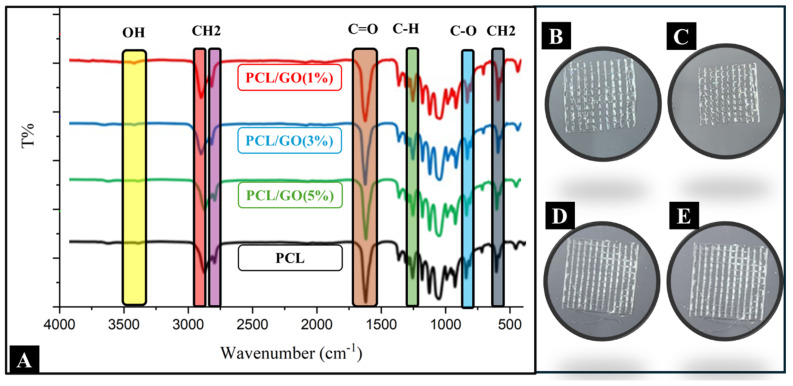
(**A**) FTIR spectrums of PCL and PCL/GO meniscus scaffolds with varying GO concentrations (1%, 3%, and 5%), (**B**) PCL 3D printed (12 layers), (**C**) PCL/GO (1%) 3D printed (12 layers), (**D**) PCL/GO (3%) 3D printed (12 layers), (**E**) PCL/GO (5%) 3D printed (12 layers).

In the spectrum (Figure 4A), a broad peak belonging to hydroxyl (OH) groups was observed in the range of 3500–3200 cm^−1^ [37,38]. The intensity increase in this region indicates the contribution of OH groups in the structure of GO. This indicates the successful incorporation of GO into the PCL matrix. In the 2950–2850 cm^−1^ range [39], symmetric and asymmetric stretching vibrations of the characteristic methylene (CH_2_) groups of PCL scaffold (Figure 4B) were clearly detected. Despite the addition of GO in different percentages, no significant position change was observed in the bands in this region. However, slight increases in the intensity of the bands are observed parallel to the amount of GO. In PCL/GO (1%) (Figure 4C), PCL/GO (3%) (Figure 4D), and PCL/GO (5%) (Figure 4E) composites, CH_2_ groups became more prominent. This suggests that besides the interactions of GO with PCL, methylene groups are more present in the structure of the polymer. In the 1720–1740 cm^−1^ range [40], a strong peak belonging to the carbonyl (C=O) groups of PCL was observed. These peaks of carbonyl groups maintained their position in the presence of GO and showed only small intensity changes. This indicates that GO has weak physical or chemical interactions with PCL. The range 1150–1250 cm^−1^ [41] represents the C-O stretching vibrations of the ester bonds in the PCL structure. Despite the contribution of GO, no significant position change was detected in this region, suggesting that GO does not affect ester bonds. In the 500–1000 cm^−1^ range [39,42], CH_2_ oscillatory vibrations of PCL- and GO-specific bands were observed. It was determined that the band intensities increased in this region with an increasing GO ratio. This indicates that GO is homogeneously dispersed in the PCL matrix and the composite structure maintains its integrity. As a result, FTIR analysis revealed that the incorporation of GO into the PCL structure did not lead to a significant change in chemical bonding but caused differences in properties such as increased intensity in the bands belonging to OH groups. These findings reveal that the interactions of GO with PCL occur at the physical level and the chemical structure of PCL is preserved, but the incorporation of GO improves the properties of the composite material.

### 4.6. Swelling and Weight Loss Rate

**Figure 5:**
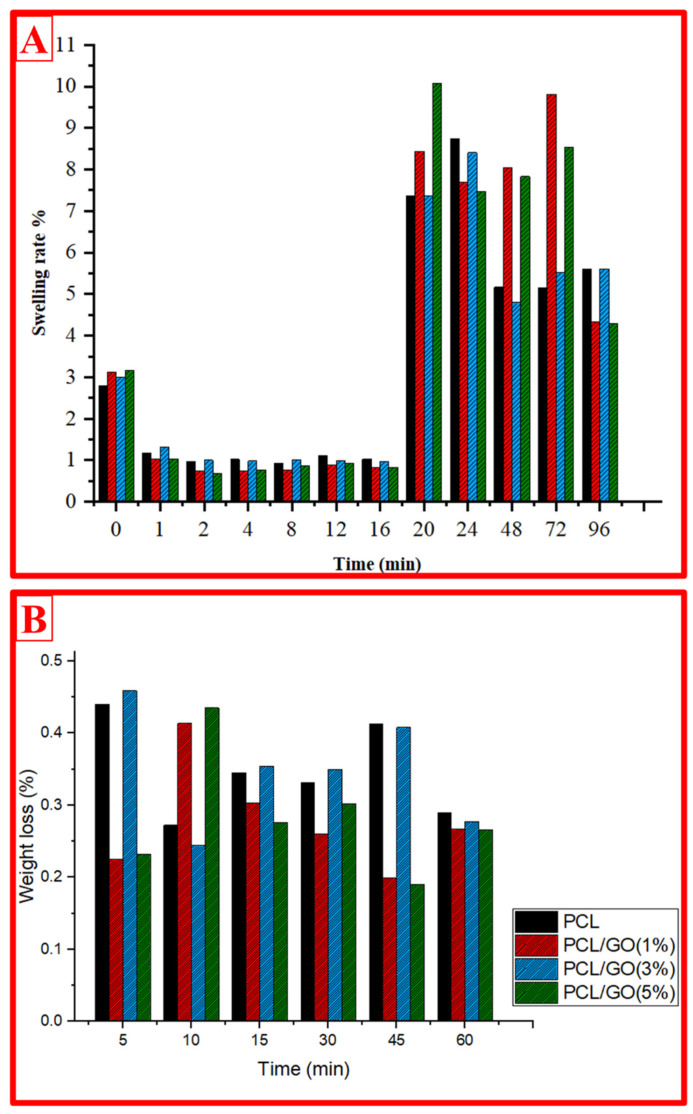
Swelling (**A**) and degradation (**B**) behaviors of the 3D-printed scaffolds.

The swelling behavior of PCL and PCL/GO composites (1%, 3%, and 5%) is expressed as a function of time (Figure 5A). Initially, the swelling rate of the PCL scaffold remains at lower levels compared to the GO-reinforced composite scaffolds. This can be interpreted as GO increases the water absorption capacity due to its hydrophilic nature. A significant swelling increase is observed in all samples, especially from 24 min onwards, and reaches a plateau level at 72–96 min. However, as the percentage of GO increases, the increase in the swelling rate becomes more pronounced. This indicates that the homogeneous distribution of GO in the polymer matrix and the hydrogen bonds formed between GO and polymer attract water more effectively [43,44].

It was also observed that the composite scaffolds containing 1% and 3% GO reached a higher swelling ratio compared to the sample containing 5% GO. This suggests that if GO is used in high proportions, it may partially reduce water permeability by causing agglomeration in the matrix. Overall, these results reveal that GO as a hydrophilic additive can optimize the swelling properties [44,45,46].

Figure 5B examines weight loss as a function of time and reflects the degradation behavior of the samples. The weight loss of the PCL scaffold is generally higher than that of the GO-doped composite scaffolds. This indicates that GO enhances the mechanical stability and thermal durability of the polymer matrix. In particular, composites containing 3% and 5% GO show lower weight loss in long-term tests. This can be explained by the fact that GO provides a barrier effect, limiting the interaction of the polymer structure with water and thus slowing down the degradation process [47,48,49]. When analyzed as a function of time, an increasing trend in weight loss is observed, especially in the first 10 min. This may be due to the initial dissolution of the water contact areas on the polymer surface. However, in longer tests, this trend slows down and reaches a constant level. This process slows down even more with increasing GO concentrations. In particular, the weight loss of the composite containing 5% GO is the lowest, indicating that this sample may be the most suitable option for long-term durability and stability in biomedical applications. In conclusion, these graphs show that GO-doped PCL composites increase the usability in biomedical applications by affecting both swelling and weight loss behaviors. Future studies could focus on evaluating the performance of such materials in different biological environments. This provides an important basis for the development of biomimetic structures and the expansion of their application areas.

### 4.7. Antibacterials Result

**Figure 6:**
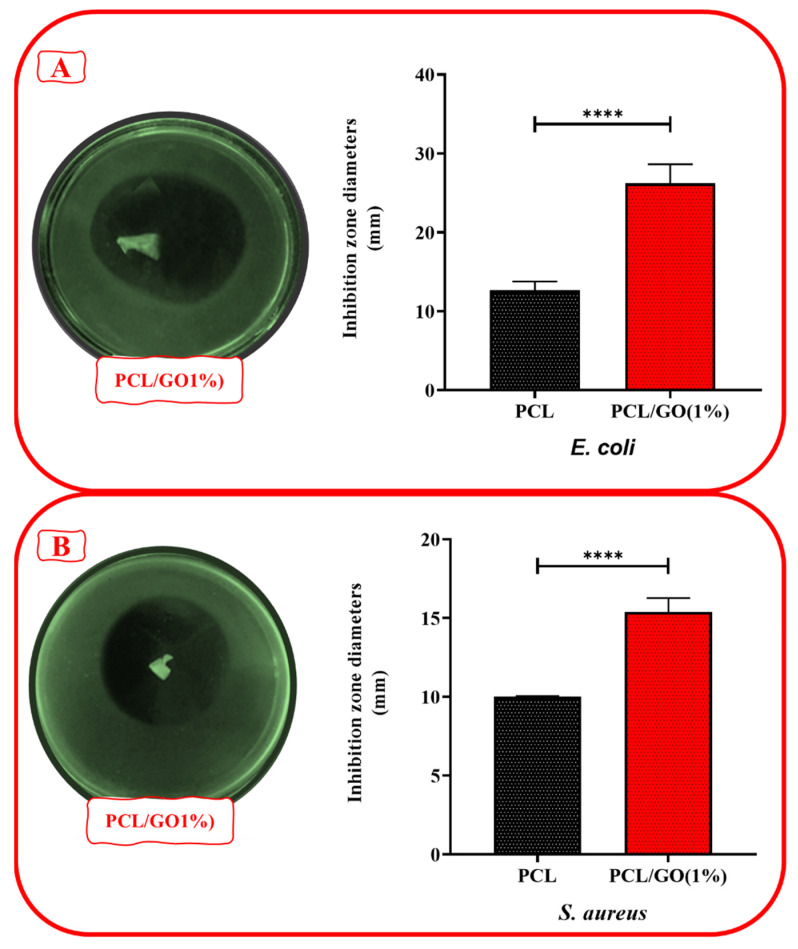
Assessment of antimicrobial effectiveness for tested 3D meniscus scaffolds via agar disc diffusion methods, respectively, by recording inhibition zones. (**A**) *E. coli*, (**B**) *S. aureus* (**** *t* test, GraphPad; the statistical significance level was determined as *p* < 0.0001).

The PCL/GO (1%) scaffold showed a pronounced antibacterial effect on *E. coli* (Figure 6A). The 26.21 mm inhibitory zone was much larger than the 12.65 mm zone provided by PCL alone. GO was observed to potentiate the antibacterial properties of PCL. This showed that it provides a much more effective solution against Gram-negative bacteria. This is because GO’s surface properties and load-carrying capacities can enhance antibacterial properties by acting on the bacterial cell wall. The PCL/GO (1%) combination also showed a stronger effect on *S. aureus* (Figure 6B), but this effect was not as pronounced as against *E. coli*. The inhibitory zone of 15.38 mm is larger than the PCL effect of 10.01 mm. Similar studies have shown that GO into PCL scaffolds improves their antimicrobial properties. In one study, PCL incorporated with reduced graphene oxide (rGO) was found to exhibit potent antibacterial activity against both Gram-positive (*S. aureus*) and Gram-negative bacteria (*E. coli*), and higher concentrations of rGO-enhanced bacteriostatic effects [50]. In another study, electroactive PCL scaffolds with thermally reduced graphene oxide (TrGO) showed complete eradication of *S. aureus* under electrical stimulation, highlighting the potential of combining electroactive and antibacterial properties for enhanced tissue regeneration [51]. These findings support the idea that the incorporation of GO-doped PCL scaffolds not only supports tissue regeneration but also mitigates bacterial growth, which is crucial for preventing infections in medical applications like meniscus implants.

### 4.8. Cytotoxicity Tests

**Figure 7:**
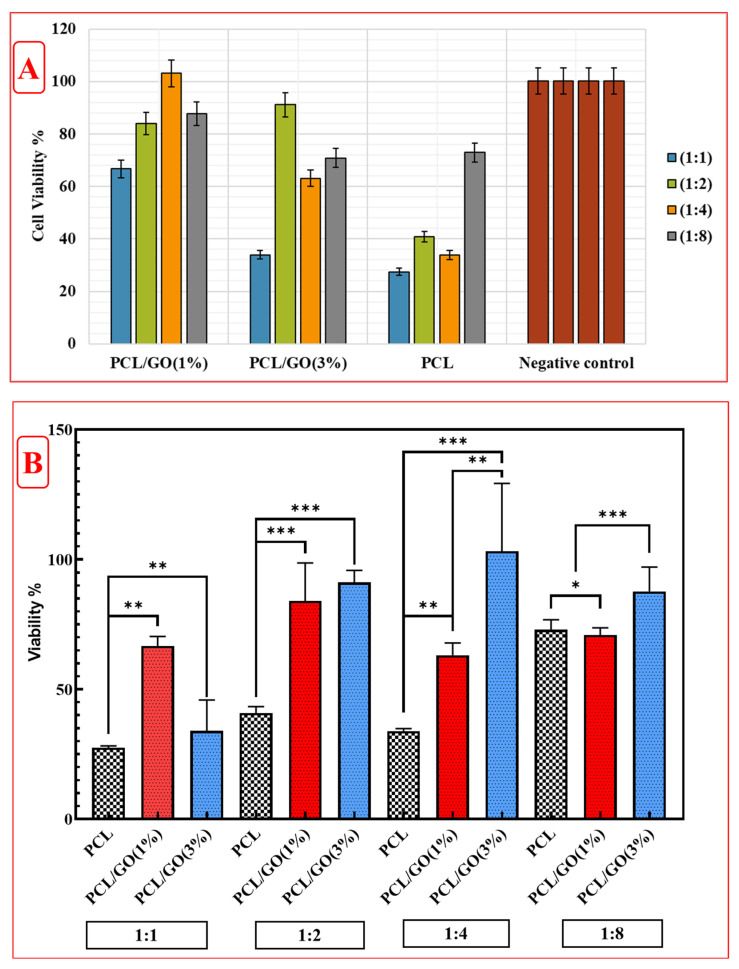
Cytotoxic activity of PCL and PCL/GO scaffolds. (**A**) L929 cells were treated for 24 h with four different concentrations of sterile samples: (1:1), (1:2), (1:4), and (1:8) via the indirect MTT method. (**B**) Cell viability of L929 cells after cytotoxicity tests of 3D composite scaffolds (*, **, *** the statistical significance level was determined as *p* < 0.05).

In vitro cytotoxicity of PCL and PCL/GO 3D meniscus scaffolds were investigated on the L929 fibroblast cell line using the MTT assay according to the ISO 10993-5-2009 standard [52]. The cytotoxic activity of 3D scaffolds at different concentrations on L929 cells is shown in Figure 7A. It was observed that the PCL/GO (1%) scaffold significantly affected cell viability at concentrations of 1:2, 1:4, and 1:8, exceeding 100% viability at 1:8 concentration. In L929 cells treated with 1:1 extract of PCL alone, cell viability was determined to be less than 50%. Viability was similarly determined to be less than 50% in extracts diluted at 1:2 and 1:4. In cells treated with extract diluted at 1:8, viability was higher than the others. As the GO (3%) additive ratio increased, cell viability decreased significantly at 1:1, 1:4, and 1:8 concentrations compared to the GO (1%) additive. However, at a 1:4 concentration, cell viability approached 90% compared to GO (1%). Cell viability was evaluated using the MTT assay at (1:1), (1:2), (1:4), and (1:8) concentrations of the cell culture, as presented in Figure 7B. In similar meniscal graft cytotoxicity studies, polycaprolactone/methacrylate anhydride gelatin/meniscus extracellular matrix (PCL/GelMA/MECM) 3D bioprinting scaffolds were designed. Cells cultured with PCL/GelMA/MECM scaffolds and PCL/GelMA/MECM/PDGF scaffolds proliferated faster after 4 and 7 days (*p* < 0.001), and the proliferative effect of PCL/GelMA/MECM/PDGF scaffolds was more pronounced [53]. In another study, a biomimetic meniscus scaffold was designed using PCL and meniscus fibrocartilage chondrocytes (MFCs)-loaded GelMA/MECM bioink. Cell viability, biodegradation, and tissue formation were achieved in vivo through the biomimetic meniscus scaffold, thus providing a reliable basis for its application in tissue engineering [54].

**Figure 8:**
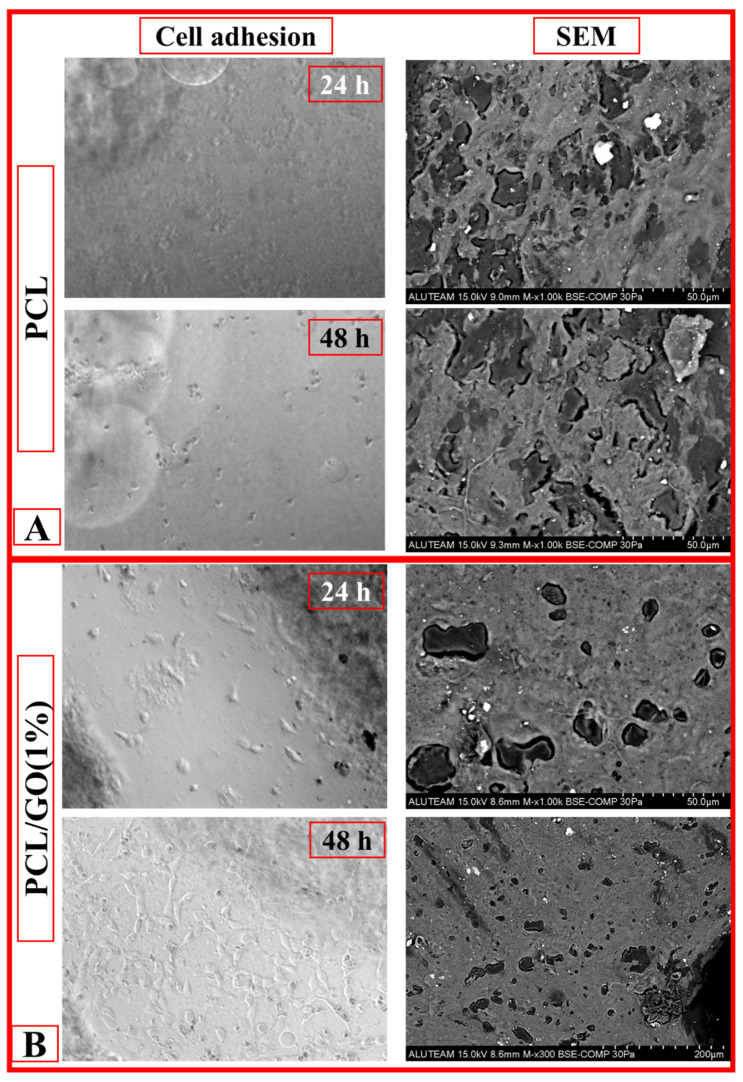
(**A**) 24 and 48 h cell adhesion–SEM images of the PCL scaffold. (**B**) 24 and 48 h cell adhesion–SEM images of the PCL/GO (1%) scaffold.

When the cell adhesion and SEM analyses on the PCL scaffold were analyzed, the spread of cells on the surface was limited. In the 24 h cell adhesion images, it is seen that the cells are irregularly distributed on the surface and do not show a widespread. In the 48 h images, it is understood that the cells spread a little more, but no obvious cellular connections and extensions were formed (Figure 8A).

It was observed that the surface became more heterogeneous and rougher with the addition of GO. It is noticed that there are more micro- and nanoscale structures on the surface, which is thought to increase the adhesion of cells. In the 24 h (Figure 8B), it was observed that fibroblast cells adhered to the surface, but the cell density was low. It was determined that the cells were scattered on the surface and had not yet entered the proliferation stage. At after 48 h (Figure 8B), it was observed that the cell density increased significantly, and a large part of the surface was covered with fibroblast cells. This indicates that the cells successfully proliferated on the scaffold and supported the biocompatibility of the scaffold. In addition, it was determined that the surface coverage rate was low at 24 h but almost completed at 48 h. This indicated that the cells spread more on the surface and entered an active proliferation process. When the cell morphology was examined, it was determined that fibroblasts showed minimal interaction with the surface and had a round morphology at 24 h. This shows that the cells were in the initial adhesion stage. At 48 h (Figure 8B), it was observed that fibroblast cells established wider contact with the surface, developed extensions (filopodia and lamellipodia), and attached strongly to the surface. These morphological changes prove that the adaptation of the cells to the surface and their biological activity increased. It was determined that PCL/GO surface properties positively affected fibroblast cell adhesion [14,33,55,56]. It was evaluated that GO additives facilitated cell adhesion and proliferation thanks to the functional groups on the surface. It was observed that the cells interacted better with the surface, and micropores were used as cell adhesion points. When cell development was evaluated over time, it was determined that the cells were in the initial stage [54,57] at 24 h, and the spreading and proliferation processes became active at 48 h. This proves the biocompatibility of the scaffold and its compatibility with fibroblast cells.

These SEM images (Figure 8B) clearly revealed the biocompatibility of the PCL/GO meniscus scaffold with fibroblast cells. The difference between 24 and 48 h clearly showed the potential of the scaffold to promote cell adhesion and increase its biological activity. It can be said that GO-doped surfaces increase biological activity in such applications. In addition, better adaptation of cells to the surface over time also supports the biomechanical suitability of the scaffold.

**Figure 9:**
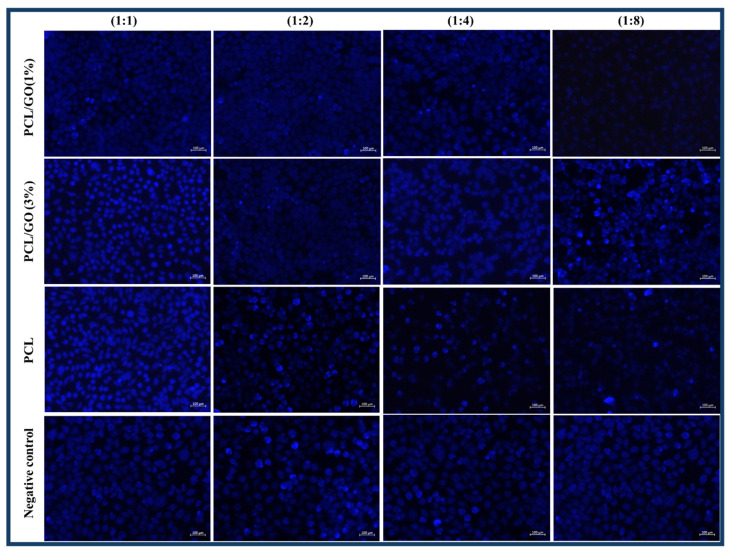
Fluorescence microscopy images of L929 cells treated with different concentrations of scaffold extract (1:1), (1:2), (1:4), and (1:8) for 24 h. After treatment, cells were stained according to the DAPI staining protocol and visualized by a fluorescence microscope.

The cell density of PCL/GO (1%) and PCL/GO (3%) 3D meniscus scaffolds was observed to be higher, especially at 1:1 and 1:2 ratios (Figure 9). This may indicate that the designed scaffolds are suitable for cell adhesion and proliferation. While generally more dense cell adhesion was observed at a 1:1 ratio, it was observed that the cell density decreased as the ratio increased (1:4, 1:8). The shape and distribution of cell nuclei were quite regular in PCL and PCL/GO scaffolds [14,58,59]. This indicated that the surface was suitable for cell adhesion. The PCL/GO (3%) meniscus scaffold performed better than other groups regarding cell density and distribution. This may indicate the positive effect of GO on biomaterials [56,59].

The structural differences or degradation of products of the scaffold may have caused such a viability profile. In comparison to GO-doped scaffolds, PCL demonstrated higher weight loss. These findings, together with the decreased cell viability at lower dilution ratios (and hence greater presence of PCL), may indicate potential toxicity caused by breakdown byproducts resulting from weight loss. The weight loss curve for GO-doped PCL scaffolds differs, and GO slows PCL breakdown. In some circumstances, the decrease in cell viability could be due to structural changes in the scaffold that prevent cell multiplication. This assumption may be applied to both GO-doped PCL scaffolds (1% and 3%), which exhibit an inhibitory trend following an initial boost in viability. It is unclear how the structure of 3D scaffolds suppresses cell proliferation and affects cell viability, but it is a source of debate in the scientific and research communities.

In addition, the viability test is usually measured based on the metabolic activity of the cells. DAPI staining shows only the nuclei. Metabolic activity may be high, but cell density may be low, in which case, viability appears high on the graph, while the number of cells may appear low in DAPI images. Fewer but more metabolically active cells may give high viability results. Therefore, it is normal for the viability tests and microscopic images not to match exactly, and both data should be interpreted together.

## 5. Conclusions

Incorporating GO-coated PCL scaffolds has demonstrated promising outcomes for meniscus cartilage regeneration. The enhanced rheological and mechanical properties, including increased viscosity and elastic moduli, suggest that GO contributes to structural stability and mimics the mechanical characteristics of native meniscus tissue. Thermal analysis confirmed improved thermal stability, while FTIR analysis indicated the preservation of the chemical integrity of the PCL matrix. Biological evaluations showed that GO-enriched scaffolds support cell adhesion, proliferation, and viability, with significant antibacterial activity against Gram-negative and Gram-positive pathogens. Among the tested compositions, GO (1%) demonstrated a balanced profile, optimizing mechanical and biological performance. These findings suggest that the PCL/GO 3D scaffolds fabricated via 3D bioprinting are a viable candidate for use in meniscal repair and regeneration. Future studies should focus on in vivo evaluations to further assess the clinical applicability of these scaffolds.

## Figures and Tables

**Table 1:** Concentrations of PCL and PCL/GO solutions.

Weight Difference (%)				
Solutions	PCL (wt%)	GO (wt%)	GO (wt%)	GO (wt%)
PCL 20%wt	20	-	-	-
PCL/GO (1%wt)	20	(1%wt)	-	-
PCL/GO (3%wt)	20	-	(3%wt)	-
PCL/GO (5%wt)	20	-	-	(5%wt)

**Table 2:** Rheological characteristics of the PCL and PCL/GO hydrogels at room temperature with 1% strain at 1 Hz.

	G′ (Pa)	G″ (Pa)	Shear-Thinning Viscosity (Pa·s)	Shear Rate (s^−1^)	Yield Shear Stress (Pa)
PCL	36.1 ± 2.13	8.7± 0.19	19.1 ± 1.22	8.7	97.2 ± 8.09
PCL/GO (1%wt)	48.5 ± 1.12	14.6 ± 1.33	39.5 ± 0.13	14.1	308.4 ± 4.17
PCL/GO (3%wt)	59.1 ± 0.25	23.7 ± 2.11	50.9 ± 4.04	26.3	385.2 ± 2.09
PCL/GO (5%wt)	97.1 ± 0.08	34.4 ± 1.04	89.3 ± 1.17	49.8	507.1 ± 3.63

**Table 3:** Elastic modulus, ultimate stress and strain at break of 3D PCL and PCL/GO meniscus scaffolds.

	E modulus (MPa)	Ultimate Stress (MPa)	Strain at Break (%)
**PCL**	635.3	45.3	9.2
**PCL/GO (1%)**	614.1	46.3	9.9
**PCL/GO (3%)**	592.1	43	9.3
**PCL/GO (5%)**	588	46	10.4

## Data Availability

The authors confirm that the data supporting the findings of this study are available within this article. Raw data that support the findings of this study are available from the corresponding author, upon reasonable request.
